# A Herpesvirus Specific Motif of Epstein-Barr Virus DNA Polymerase Is Required for the Efficient Lytic Genome Synthesis

**DOI:** 10.1038/srep11767

**Published:** 2015-06-30

**Authors:** Yohei Narita, Atsuko Sugimoto, Daisuke Kawashima, Takahiro Watanabe, Teru Kanda, Hiroshi Kimura, Tatsuya Tsurumi, Takayuki Murata

**Affiliations:** 1 Division of Virology, Aichi Cancer Center Research Institute, 1-1 Kanokoden, Chikusa-ku, Nagoya 464-8681, Japan; 2 Department of Virology, Nagoya University Graduate School of Medicine, 65 Tsurumai-cho, Showa-ku, Nagoya 466-8550, Japan; 3 Division of Microbiology and Oncology, Aichi Cancer Center Research Institute, 1-1 Kanokoden, Chikusa-ku, Nagoya 464-8681, Japan

## Abstract

Epstein-Barr virus (EBV) is associated with several malignancies, including Burkitt lymphoma and nasopharyngeal carcinoma. To overcome such disorders, understanding the molecular mechanisms of the EBV replication is important. The EBV DNA polymerase (Pol) is one of the essential factors for viral lytic DNA replication. Although it is well known that its C-terminal half, possessing DNA polymerase and 3’-5’ exonuclease activity, is highly conserved among Family B Pols, the NH_2_-terminal half has yet to be characterized in detail. In this study, we show that a stretch of hydrophobic amino acids within the pre-NH_2_-terminal domain of EBV Pol plays important role. In addition, we could identify the most essential residue for replication in the motif. These findings will shed light on molecular mechanisms of viral DNA synthesis and will help to develop new herpesviruses treatments.

The Epstein-Barr virus (EBV) is a human γ-herpesvirus that mainly infects and establishes latent infection in B lymphocytes, but it also can infect other types of cells, such as NK, T and epithelial cells. EBV is the most common cause of infectious mononucleosis and is also associated with several malignancies, including Burkitt lymphoma and nasopharyngeal carcinoma. EBV can take either a latent infection or a lytic productive stage in infected cells^1^,^2^. The EBV genome persists as a nuclear episome, a circular plasmid molecule, during the latent infection and replicates once in S phase using cellular DNA replication machinery. However, a small percentage of infected cells switch from the latent stage into the lytic cycle, which is triggered by expression of an immediate-early protein, BZLF1, to produce progeny viruses.

EBV encodes seven proteins that are essential for the lytic genome replication, these being the oriLyt binding protein (BZLF1), the single-stranded DNA (ssDNA) binding protein (BALF2), the heterotrimeric helicase/primase complex (BSLF1, BBLF2/3 and BBLF4), the DNA polymerase (Pol) (BALF5), and its processivity factor (BMRF1). After induction of productive viral replication, the EBV genome is amplified 100- to 1,000-fold by viral replication machinery in discrete sites in nuclei, called replication compartments^3^,^4^,^5^. EBV Pol BALF5 possesses intrinsic 5’-to-3’DNA polymerase and 3’-to-5’ exonuclease activities that are characteristic functions of the replicative Pol B family^6^ and forms heterodimers with the BMRF1 polymerase accessory protein to exhibit high polymerase processivity^7^. Unlike the case of the eukaryotic replication apparatus, the EBV DNA Pol holoenzyme is used in the synthesis of both leading and lagging strands at replication forks^6^. The C-terminal half of herpesvirus Pol has been well studied, especially in herpes simplex virus UL30, the region bearing 5’-3’ polymerase and 3’-5’ exonuclease activities. Unlike the C-terminus, functions of the NH_2_-terminal half have not been investigated in detail. We previously found that peptidyl-prolyl *cis*-*trans* isomerase NIMA-interacting 1 (Pin1) interacts with BALF5 through NH_2_-terminal pThr178-Pro179, and may modulate the EBV Pol conformation for efficient, productive DNA replication^8^. This result implies that the NH_2_-terminal portion of EBV Pol is also important, presumably for the protein folding and maintenance of the 3-D structure. Crystal structure analysis of HSV Pol revealed two domains at the NH_2_-terminus: NH_2_-terminal and pre-NH_2_-terminal^9^. The NH_2_-terminal domain consists of three structural motifs that have similarities to NH_2_-terminal domains of family B polymerases. On the other hand, the pre-NH_2_-terminal domain is present at the extreme NH_2_-terminus of herpesvirus Pols. Recently, a conserved FYNPYL motif at the residues 44 to 49 of HSV-1 Pol in the pre-NH_2_-terminal domain was found to play an important role in viral DNA synthesis and production of infectious viruses *in vitro*^10^ and *in vivo*^11^. Significance of the conserved motif of HSV-1 Pol indicates that the pre-NH_2_-terminal domain of herpesvirus Pols likely possesses unknown function that affects genome synthesis efficiency and production of progeny viral particles. Since EBV Pol also has the conserved sequence (FYNPFL) in its pre-NH_2_-terminal domain, we here focused on the hydrophobic sequence, and confirmed that this motif plays a pivotal role in EBV productive replication.

## Results

### The pre-NH_2_-terminal conserved motif of EBV DNA Pol is involved in lytic genome replication

**Figure 1:**
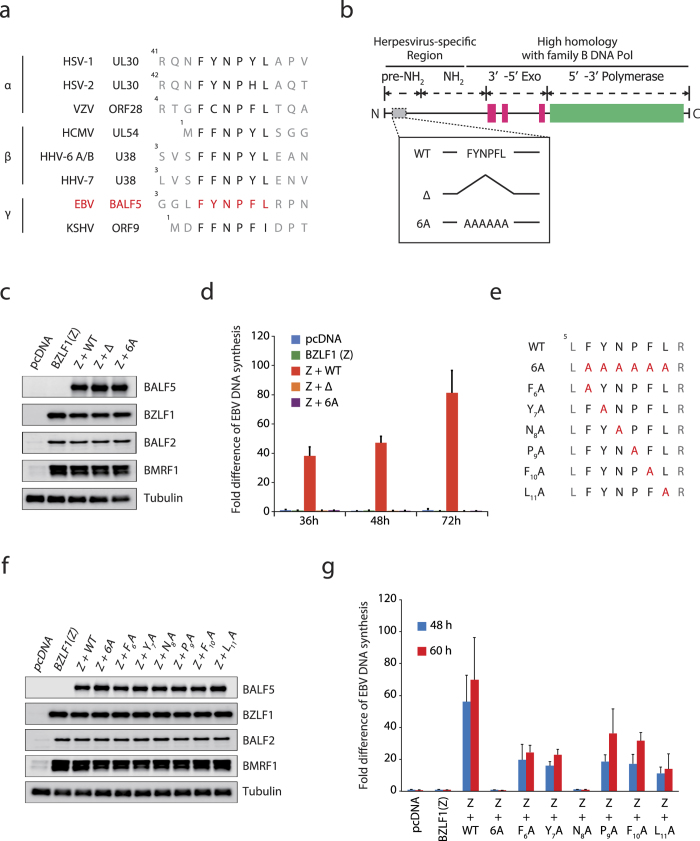
Mutation in the conserved motif of Pol inhibits viral lytic genome amplification. (**a**) Sequence alignment of eight human herpesvirus Pol sequences^10^. The hydrophobic and aromatic residues (FYNPFL) 6 to 11 of EBV Pol are highly conserved in the human herpesvirus Pol family. Polymerase sequences of herpes simplex virus type 1 (HSV-1)^26^,^27^, HSV-2^28^, varicella-zoster virus (VZV)^29^, human cytomegalovirus (CMV)^30^, human herpesvirus 6 (HHV-6)^31^, HHV-7^32^, Epstein-Barr virus (EBV)^33^, and Kaposi’s sarcoma-associated herpesvirus (KSHV)^34^ are shown with each Pol name. (**b**) The scheme of BALF5 mutants used in this paper. Relative positions of conserved motif, exonuclease (Exo) domain and polymerase (Pol) domain are shown respectively. (**c**) Protein expression levels 24 h after transfection of BZLF1 and/or a Pol expression vector. (**d**) Relative levels of EBV genome at 36, 48, and 72 h after transfection. Each bar represents the mean and standard deviation for the viral DNA level after normalization, calculated from three independent samples. (**e**) Alignment of a series of BALF5 point mutants. The substituted residues are highlighted in red. (**f**) Viral/cellular protein expression levels. Expression vectors were transfected into HEK293EBV-BAC BALF5Δ cells as indicated at the top. (**g**) Remaining cells were lysed at 48 and 60 h post-transfection, and EBV genome levels were measured by qPCR. Each bar represents the mean and standard deviation for the viral DNA level after normalization, calculated from three independent samples.

While the C-terminal half of herpesvirus DNA Pol is well conserved in terms of sequence homology and alignment of functional domains, most of the NH_2_-terminal portion is relatively less conserved. However, Terrell and Coen recently reported that the FYNPYL motif in the pre-NH_2_-terminal domain of HSV-1 Pol (UL30) is required for efficient viral DNA replication^10^. This result indicated significance of the NH_2_-terminus of the Pol for virus genome replication. Since this hydrophobic motif is highly conserved among all members of the human herpesvirus DNA Pol family (Fig. 1a)^10^, we hypothesized that the EBV-encoded DNA Pol (BALF5) was also regulated in the same manner as the HSV-1 Pol. In order to investigate the importance of the pre-NH_2_-terminal conserved motif, we constructed two expression vectors expressing EBV DNA Pol mutants: a truncation mutant BALF5 Δ and an alanine substituted mutant BALF5 6A (Fig. 1b). To determine whether mutations in the motif of BALF5 affect virus genome replication, cell-based replication assays were carried out (Fig. 1c,d), using HEK293 cells latently infected with the BALF5-deleted EBV-BAC (HEK293EBV-BAC BALF5Δ), generated previously^8^. In the BALF5 knockout EBV cell line, exogenous expression of BZLF1 led to induction of early genes, such as BALF2 and BMRF1, but not BALF5 protein (Fig. 1c). Because of the lack of BALF5, the DNA Pol catalytic subunit, the virus could not amplify viral DNA even after induction with BZLF1 (Fig. 1d). Exogenous supply of wild type of BALF5 could restore viral DNA synthesis, while truncated and substituted mutants were almost incapable of increasing viral DNA levels (1.23% and 1.25%, respectively) (Fig. 1d, Supplemental Table 3a).

### The Asn residue in the conserved motif plays a key role in viral lytic genome replication

We next tried to identify the principal amino acid residue that contributes to viral genome replication by cell-based replication assay. As shown in Fig. 1e, we constructed several vectors expressing mutants in which each amino acid was substituted with alanine. Then, the expression vector for BALF5 wild type (WT) or its mutants were transfected to HEK293EBV-BAC BALF5Δ cells with the BZLF1 expression vector. At 24 h post-transfection, BALF5 protein expression levels of any of the substituted mutants were mostly equivalent to that of the wild type of BALF5 (Fig. 1f). As shown in Fig. 1g, when the EBV DNA Pol (BALF5 WT) was *trans*-complemented, the amount of viral DNA increased up to 60–80 fold. Interestingly, single amino acid substitution at any of the residues in the motif attenuated virus genome replication (20.2% ~ 35.2% of Z+WT) (Fig. 1g, Supplemental Table 3b), but importantly, substitution of the conserved asparagine (Asn) residue at the 8th position with alanine almost completely eliminated EBV genome synthesis (2.05%) (Fig. 1g, Supplemental Table 3b). The viral DNA level with BALF5 N_8_A was almost comparable to negative controls, suggesting that the Asn residue most profoundly contributes to DNA pol catalytic activity.

### Mutations at the conserved motif did not affect the polymerase catalytic activity *in vitro*

**Figure 2:**
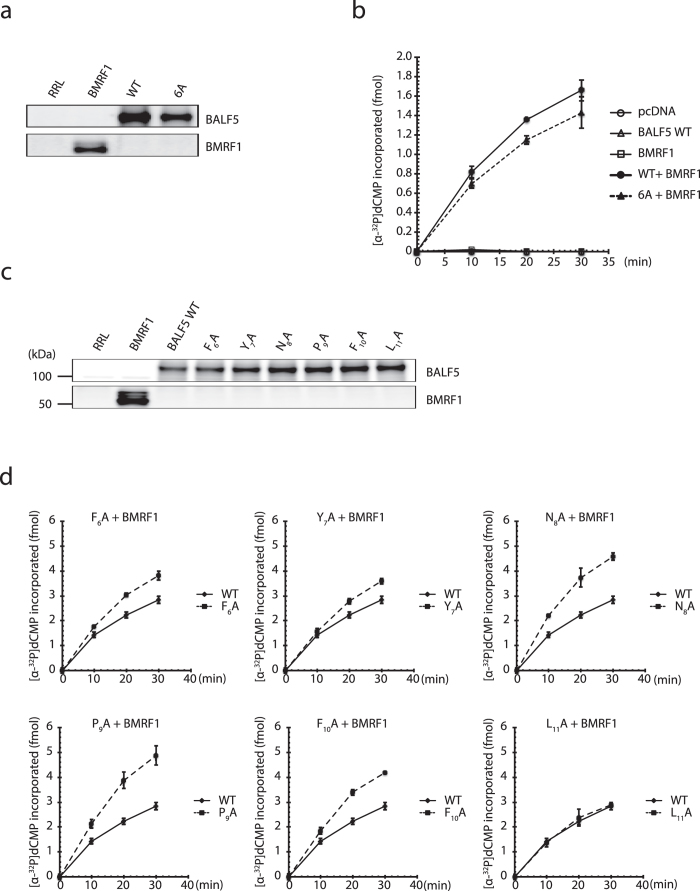
Mutation in the conserved motif does not affect polymerase enzymatic activity *in vitro*. (**a**) The amount of each protein was confirmed by immunoblotting, using the antibodies indicated on the right. RRL; rabbit reticulocyte lysate. (**b**) The total amount of [α-^32^P]dCMP incorporated during the incubation period (fmol) is shown. (**c**) Protein expression levels of each mutant Pol were checked by immunoblotting. (**d**) Polymerase enzymatic activity of each mutant Pol protein was assessed.

To extend these findings, we then carried out the *in vitro* polymerase assays. EBV Pol (BALF5) and processivity factor (BMRF1) proteins were prepared in Rabbit reticulocyte lysate (RRL) with an *in vitro* transcription/translation system^10^,^12^,^13^. To assess background activity of endogenous RRL proteins, empty vector pcDNA was used as a control. *In vitro* polymerase assays were performed by measuring the incorporation rate of [α-^32^P]dCMP into activated calf thymus DNA, followed by dot blotting to anion exchange paper, washing, and quantitative autoradiography. In order to reduce background polymerase activities, while preserving EBV DNA Pol activity, 100 mM of ammonium sulfate was added in the reaction buffer^14^,^15^. Proteins expressed in the RRL were confirmed by western blotting (Fig. 2a).

Under this condition, EBV Pol BALF5 alone could not enhance incorporation of nucleotides, and resulted in background levels. On the other hand, with addition of BMRF1 protein, the polymerase processivity factor, EBV Pol exhibited its full activity and efficiently incorporated nucleotides (Fig. 2b). We also checked here that BMRF1 protein alone was incapable of synthesizing DNA. Interestingly, mutation of the conserved motif did not significantly inhibit the polymerase activity (Fig. 2b). These results indicate that mutations in the pre-NH_2_-terminal conserved motif do not affect the Pol enzymatic activity itself, and also suggest that EBV Pol protein may be regulated in a complicated manner in cells that could not be reflected under our *in vitro* DNA incorporation system, at least. Similarly, we tried to check *in vitro* catalytic activity of the single amino acid mutant Pols used in Fig. 1e–g. All of the mutants could incorporate [α-^32^P]dCMP as efficiently as wild-type BALF5 (Fig. 2c,d). These results indicate that the pre-NH_2_-terminal hydrophobic residues of EBV Pol, especially the Asn_8_, are important for lytic replication of the virus, but site-directed mutagenesis of the residues did not abolish catalytic activity of the Pol.

### Mutant recombinant virus failed to synthesize viral DNA and produce infectious virions efficiently

**Figure 3:**
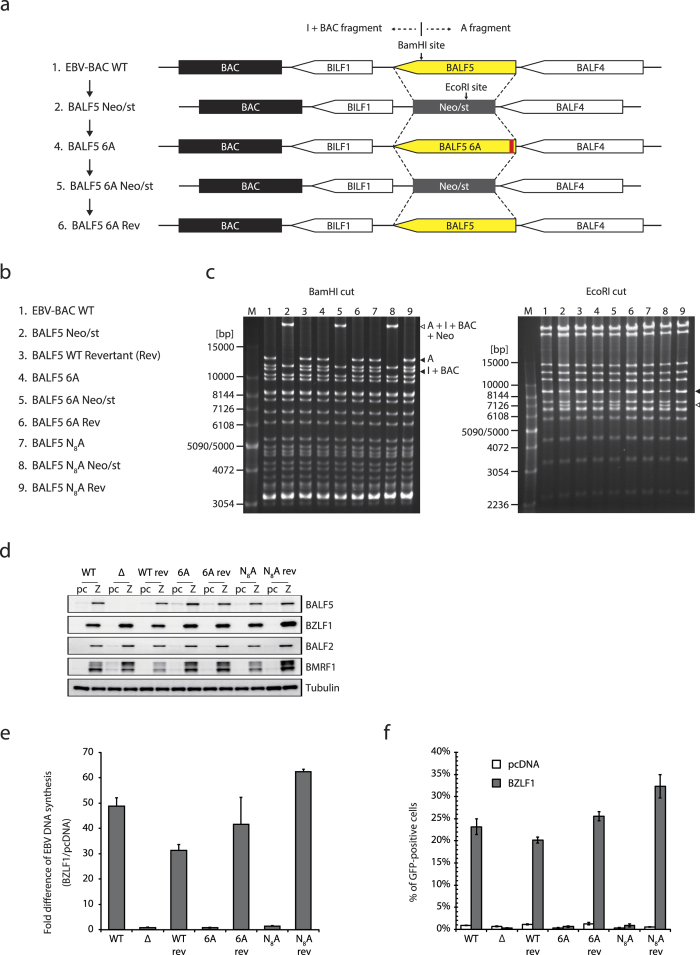
Significance of the conserved motif in the context of virus genome. (**a**) Schematic arrangement of the recombination of the EBV-BAC genome to obtain BALF5 6A and its revertant (Rev). The whole sequence of BALF5 open reading frame^35^ was replaced with the neomycin-resistance and streptomycin-sensitivity genes (Neo/st) cassette. The cassette was then replaced to BALF5 sequence with 6A mutation to obtain mutant BALF5 6A. Likewise, mutant Pol was replaced with Neo/st cassette, and then the mutation was restored to wild type (BALF5 6A Rev). Schemes for other mutant viruses are shown in Supplemental Figure 1. Approximate positions of BamHI and EcoRI site were indicated. (**b**) The list of EBV-BAC clones we generated. (**c**) Electrophoresis of the recombinant viruses. The recombinant EBV genomes were digested with either BamHI or EcoRI and separated in an agarose gel. The numbers above each panel correspond to that of (**b**). A, the A fragment of BamHI-digested EBV-BAC. I, the I fragment of BamHI-digested EBV-BAC. (**d**) Each HEK293 EBV-BAC cell clone was transfected with 50 ng of empty vector or BZLF1 expression vector using a Microporator (Digital Bio). Aliquots of cells were harvested at 24 h after transfection and subjected to immunoblotting with indicated antibodies. pc, pcDNA. Z, BZLF1. (**e**) The remaining cells were harvested at 36 h after transfection and subjected to qPCR. Each bar represents the mean and standard deviation for the viral DNA level after normalization, calculated from three independent samples. (**f**) Culture supernatants from HEK293 EBV-BAC cells, transfected in the same fashion as described for panel d and e, and followed by 4 days of incubation, were collected and used to infect naive Akata(−) cells. Viral load in the medium was determined by fluorescence-activated cell sorting analysis and is shown as the percentage of GFP-positive cells.

Our experiments so far showed that the pre-NH_2_-terminal conserved motif mutant of EBV DNA Pol could not amplify the viral genome efficiently in cell-based complementation assay. In order to check whether the results obtained from *trans*-complementation assays reflects the original nature of EBV lytic genome replication machinery, we generated several recombinant EBV with the motif mutation. Figure 3a depicts strategy of preparing BALF5 6A mutant and its revertant strain BALF5 6A Rev. Schemes for preparation of other recombinant viruses are shown in Supplemental Figure 1. We prepared a total of 9 BAC clones as listed in Fig. 3b. Because BALF5 ORF and Neo/st cassette possess BamHI and EcoRI site, respectively (Fig. 3a), integrity of the BAC DNA was checked by either BamHI and EcoRI digestion, followed by electrophoresis to confirm that the recombinant viruses did not carry obvious deletions or insertions (Fig. 3c).

Recombinant EBV-BAC DNA was introduced into HEK293, as described previously^8^ and we obtained several BALF5 mutant EBV-BAC cells. To assess the levels of EBV lytic genome amplification, cells were transfected with either empty vector or BZLF1 expression vector. Protein expression levels were checked after 24 h post-transfection (Fig. 3d). At 48 h, aliquots of cells were harvested and subjected to qPCR assay (Fig. 3e). As expected from the results of *trans*-complementation assays (Fig. 1d,g), both BALF5 6A and N_8_A mutant failed to amplify viral genome DNA efficiently (1.59% and 2.97%, respectively, Supplemental Table 3c). We also measured infectious viral particles produced from the cells (Fig. 3f). At 96 h post-transfection, cells and media were harvested, freeze-thawed, and incubated with naive Akata(−) cells. Since the recombinant EBVs used here encode green fluorescent protein (GFP), Akata(−) cells infected with EBV become GFP positive. Two days after infection, cells were fixed and analyzed by FACS. When BALF5 6A or N_8_A mutant was transfected with BZLF1, less than 1% of infectious particles were obtained on average (Fig. 3f). On the other hand, wild type and revertant viruses efficiently produced infectious virions (approximately 20-30% of GFP-positive cells on the average). We conclude, from these observations, the conserved motif of EBV Pol is required for efficient viral DNA synthesis and production of progeny virus.

### The BALF5 6A mutant of EBV DNA Pol fails to form viral replication compartments

**Figure 4:**
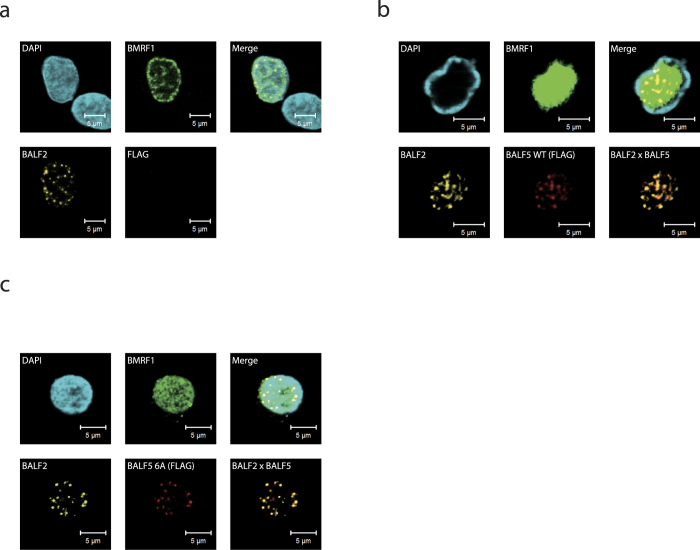
Mutation in the motif of Pol does not alter its localization to BALF2/BALF5 foci. (**a**) HEK293EBV-BAC BALF5Δ cells, transfected with the BZLF1 expression vector, were fixed and stained with DAPI (blue), anti-BMRF1 (green) and anti-BALF2 (yellow) antibodies, and observed by laser scanning confocal microscopy. The upper right panel is a merged image. (**b** and **c**) Images of HEK293EBV-BAC BALF5Δ cells, transfected with BZLF1 and FLAG-BALF5 WT (**b**) or FLAG-BALF5 6A vectors (**c**). Cells were fixed and stained with DAPI (blue), anti-BMRF1 (green), anti-BALF2 (yellow), and anti-FLAG (red) antibodies, and examined by laser scanning confocal microscopy. The upper right image is a merged image, and the lower right panel presents a merged image of BALF2 and FLAG-BALF5.

Since the pre-NH_2_-terminal conserved motif proved essential for efficient lytic replication of EBV in our cell-based assays (Figs 1 and 3), while the motif was not needed for catalytic activity when assessed under simple conditions (*in vitro*) (Fig. 2), we considered the conserved motif might play a role in its localization in cells. We previously reported that EBV Pol, not having a nuclear localization signal, cannot be transported to the nucleus when singly expressed, and that co-expression of a viral processivity factor, BMRF1, results in nuclear targeting of the Pol, with the support by HSP90^16^. In order to assess whether the mutation in the pre-NH_2_-terminal conserved motif alters protein localization, we tried to visualize the protein localization of BALF5, BALF2 and BMRF1 by three-colored indirect immunofluorescence (Fig. 4). HEK293EBV-BAC BALF5Δ cells were transfected with expression vector for FLAG-tagged BALF5 wild type (FLAG-BALF5 WT) or FLAG-BALF5 6A, together with the BZLF1 expression vector to induce lytic replication. After 48 h, cells were harvested and extracted with 0.5 % Triton X-100 mCSK buffer to extract soluble viral or cellular proteins for investigation of remaining DNA-bound fractions. This method can efficiently and clearly identify the fine structure of replication compartments in EBV-infected cells^3^,^17^.

When cells were transfected with BZLF1 expression plasmid alone, BMRF1 protein was detected in the nucleus, but the BMRF1 core, the reservoir of synthesized viral DNA that is formed by edging out the host genome DNA to the periphery^17^, could not be formed (Fig. 4a) because EBV in this cell line is devoid of BALF5. However, BALF2 proteins were detected as discrete foci in the nuclei as previously observed in the presence of phosphonoacetic acid, a specific inhibitor of herpesvirus Pols^3^.

On the other hand, when FLAG-tagged BALF5 WT was expressed with BZLF1, BMRF1 cores were readily formed, displacing host genome to the nuclear periphery (Fig. 4b, DAPI). FLAG-BALF5 WT appeared as discrete foci in the nucleus, co-localizing with BALF2, the single stranded DNA-binding protein (Fig. 4b). Such foci, composed of BALF2 and BALF5 co-localizing in the nucleus, are considered to be places where lytic replication of the genome is ongoing^3^,^17^. BALF2/BALF5 foci and BMRF1 core structures are two typical images we observe when efficient viral DNA amplification takes place.

When FLAG-BALF5 6A was tested, the BMRF1 proteins were uniformly present in the nucleus, not forming BMRF1 cores (Fig. 4c, DAPI, BMRF1). The mutant Pol co-localized with BALF2 protein, as efficiently as the FLAG-BALF5 WT, forming scattered foci in the nucleus (Fig. 4c). Thus, 6A mutant Pol can be recruited to sites where viral DNA replication starts in the nucleus and appears to form prereplication complexes, but *de novo* replication of viral DNA does not happen. Therefore, we speculate viral genome synthesis is inhibited by another mechanism besides the process of BALF5 localization to the replication factory.

### The mutant EBV DNA Pol can associate with viral replication proteins

**Figure 5:**
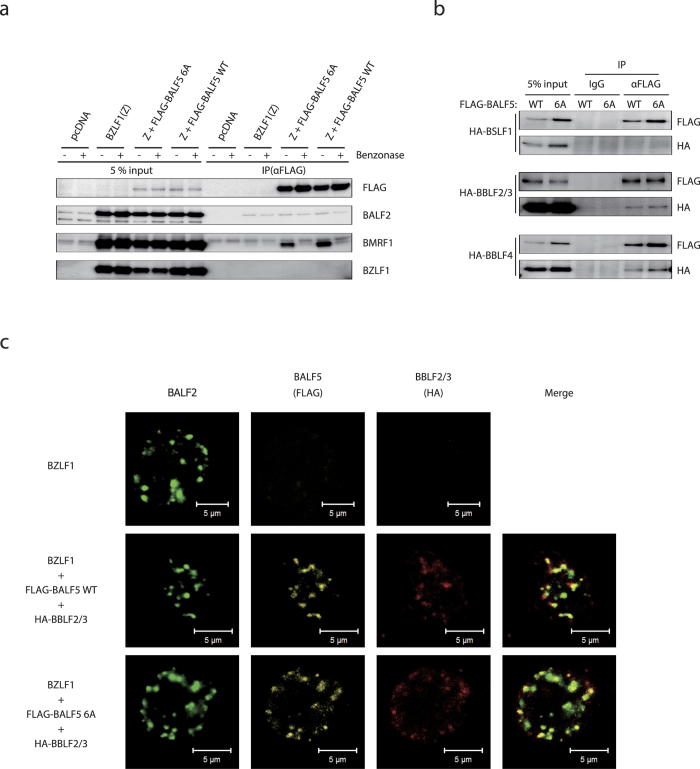
Interaction of FLAG-BALF5 WT and 6A with EBV replication proteins. (**a** and **b**) HEK293EBV-BAC BALF5Δ cells were transfected with expression vectors for BZLF1 and FLAG-BALF5 wild type (WT) or 6A, with or without the expression vector for HA-tagged EBV replication proteins, as indicated. Cell proteins were lysed and incubated with (+) or without (−) Benzonase, then subjected to immunoprecipitation using anti-FLAG M2 antibody. The lysates (5% input) and precipitates (IP) were then immunoblotted using antibodies as indicated. (**c**) HEK293EBV-BAC BALF5Δ cells, transfected with expression vectors as noted (left of panels), were fixed and stained with anti-BALF2 (green), anti-FLAG (yellow) and anti-HA (red) antibodies, and observed by laser scanning confocal microscopy.

We next wondered whether the motif might be needed for association between the Pol and viral replication complexes. To explore the possibility that the pre-NH_2_-terminal hydrophobic motif might interact with viral replication factors, we performed immunoprecipitation assays. First, HEK293EBV-BAC BALF5Δ cells were transfected with BZLF1 and FLAG-BALF5 WT/6A, and at 36 h post-transfection, cells were harvested and lysed with IP-lysis buffer. To minimize the possibility that any associations are mediated through DNA, a nuclease (Benzonase) was added to the reagent after cytolysis. By using anti-FLAG-M2 antibody, we could successfully precipitate either WT or 6A mutant of FLAG-tagged BALF5 protein (Fig. 5a). Under our conditions, BALF5 association with BALF2 or BZLF1 could not be clearly detected anyhow. BMRF1 could readily be co-purified with either WT or mutant BALF5 when nuclease was not added into the reaction mixture, but addition of nuclease drastically reduced the association (Fig. 5a), indicating that the interaction between BALF5 and BMRF1 is stabilized when DNA molecule is present. Anyway, BALF5-BMRF1 association was not affected by the mutation in the hydrophobic motif of BALF5.

We previously reported that BALF5 interacts with the helicase/primase complex that consists of BSLF1-BBLF2/3-BBLF4 proteins^18^. Moreover, Liu *et al.* argued, taking the crystal structure of the HSV Pol into consideration, that the conserved pre-NH_2_-terminal motif might be needed for physical interaction with helicase/primase complex^9^, although no direct evidence was provided. So, either of HA-tagged BSLF1, BBLF2/3 or BBLF4 expression vectors were transfected together with expression vector for FLAG-tagged Pol (either WT or 6A) and BZLF1 in HEK293EBV-BAC BALF5Δ cells. Due to unknown reasons, expression of HA-BBLF2/3 was somehow stronger than with the other two factors (Fig. 5b). We identified BBLF2/3 (helicase-primase associating factor) and BBLF4 (primase) were co-precipitated almost equally with both FLAG-BALF5 WT and 6A mutant in the presence of nuclease (Fig. 5b). However, BSLF1 (helicase) was not detected in the FLAG precipitate.

To obtain further evidence that the BALF5 WT/6A interact with the helicase-primase complex in the nuclei of the lytic-induced cells, we performed immunofluorescence analysis. HEK293EBV-BAC BALF5Δ cells were transfected with a several pairs of expression vectors and after 48 h, cells were harvested and extracted as described above. As shown in Fig. 5c, no signals against FLAG/HA were detected when only BZLF1 expression vector was transfected (Fig. 5c). On the other hand, when FLAG-BALF5 WT/6A and HA-BBLF2/3 were co-expressed, the three EBV replication proteins, BALF2 (ssDNA binding protein), FLAG-BALF5 (Pol), and HA-BBLF2/3 (helicase-primase subunit) colocalized at the BALF2/BALF5 foci in the nucleus (Fig. 5c). These results suggest that the pre-NH_2_-terminal conserved motif is not required for interaction of BALF5 protein with at least some of the viral replication factors.

## Discussion

In this study, we demonstrated that the pre-NH_2_-terminal conserved motif of EBV DNA Pol at residue from 6 to 11 contributes to viral lytic productive DNA replication. A recent report demonstrated involvement of a conserved motif at residues 44 to 49 of HSV-1 Pol UL30 in viral DNA replication^10^, but the mechanism was not identified. We found that the conserved pre-NH_2_-terminal motif of EBV BALF5 is essential for viral genome amplification. We further identified that an Asn residue in the conserved motif was of prime importance for viral genome replication (Fig. 1). This Asn residue is conserved in all human herpesvirus Pols at the same position (Fig. 1a), and other animal herpesviruses (please see below).

Although mutation at the motif of HSV-1 Pol (UL30) decreased viral DNA replication by only one magnitude of order^10^, EBV Pol was repressed more severely by the motif mutation (Figs. 1 and 3). This result was more than we expected. Difference in induction of lytic infection between HSV and EBV may explain this; HSV can induce lytic cycle by default upon *de novo* infection^10^, but lytic EBV replication needs to be induced from latent state. Interestingly, Terrell *et al.* recently demonstrated that replication of HSV-1 with mutant Pol was severely impaired in resting cells as compared to dividing cells^11^. Because lytic EBV DNA replication is weak even in permissive, rapidly growing cells, such as HEK293, the phenotype of EBV with mutant Pol might resemble that of HSV in the resting cells. Otherwise, divergence like this between EBV and HSV Pols may simply be accounted by the difference in subfamily. In any event, importance of the pre-NH_2_-terminal hydrophobic motif is essentially conserved beyond all doubt.

The mutant EBV Pol (BALF5) could incorporate dNTPs to activated DNA, to the nick(s) and gap(s) *in vitro*, thus 5’-3’ catalytic activity of the Pol was preserved. In cell-based *trans*-complementation and immunofluorescence assays, we clearly demonstrated that the mutant Pol could be recruited to the BALF2/BALF5 foci (Fig. 4), in which viral DNA replication is about to begin but viral DNA replication did not start (Figs 1 and 3). Further, association of the mutant Pol with helicase/primase complex was not affected (Fig. 5). When BZLF1 is expressed in EBV latently infected cells, BZLF1 acts not only as a transcription factor for immediately early and early gene expression but also binds to oriLyt replication origin to recruit other viral replication proteins, such as the Pol processivity factor BMRF1 protein^19^ and the helicase-primase complex^20^, resulting in formation of an pre-replication complex at an origin. BZLF1 has so far not been reported to have unwinding activity of duplex DNA or helicase activity. Also, molecular mechanisms of how viral elongation machinery is released from BZLF1 to start DNA replication from an origin are not known. From these backgrounds, although there is no evidence, we assume that undefined factor(s) possessing activity of unwinding of parental duplex at origin may associate with the hydrophobic motif in the NH_2_-terminus of the Pol, leading to opening of an oriLyt region. Alternatively, some protein kinase(s) may interact with the motif to phosphorylate inactive pre-replication complexes to an active form, resulting in starting DNA replication by viral elongation machinery released from BZLF1 at an origin. In order to clarify positional relationship between BZLF1 and the Pol, we tried to visualize the proteins by immunofluorescence. However, this experiment was not successful, due to technical reasons. Whatever the case, identification of the unknown factor(s) that regulates viral genome amplification is now underway.

Besides human herpesviruses, other animal herpesviruses also bear a hydrophobic motif in the pre-NH_2_-terminal domain. For example, murine gammaherpesvirus-68, murine cytomegalovirus, bovine herpesvirus-1, equine herpesvirus-1 and porcine herpesvirus-1 have ^3^FYNPYL, ^8^FFNPYL, ^16^FFNPYL, ^15^FFNPFI and ^3^FFNPYL, respectively. Therefore, if the hydrophobic motif enhances viral DNA replication through association with unknown factor(s), the factor X, either viral or cellular, must also be well-conserved not only in humans but also in animals throughout. Taken together, the conserved herpesvirus-specific motif of Pol will serve as a potential and novel target for antiviral/cancer drug development, although further studies are required to clarify the underlying mechanisms.

## Methods

### Cell culture and reagents

HEK293 and HEK293 EBV-bacterial artificial chromosome (BAC) BALF5Δ cells^8^ were maintained in Dulbecco’s modified Eagle’s medium (Sigma) supplemented with 10% fetal bovine serum. HEK293EBV-BAC BALF5Δ cells were maintained with addition of 150 μl/ml of hygromycin B to the culture medium. Rabbit anti-BZLF1, -BMRF1, -BALF2, and -BALF5 antibodies were as reported previously^7^,^21^. Anti-EBV EA-D-p52/50 mouse monoclonal antibody (MAB8186) was purchased from Chemicon. Anti-FLAG (M2), Anti-α/β-tubulin (#2148) and Anti-HA (6E2) antibodies were from Sigma and Cell Signaling, respectively. Horseradish peroxidase (HRP)-linked goat antibodies to rabbit IgG were from Amersham Biosciences. Secondary goat anti-rabbit, anti-mouse, and anti-rat IgG antibodies conjugated with Alexa 488, 594, 680, and a Zenon mouse IgG labeling kit were obtained from Molecular Probes.

### Transfection and immunoprecipitation (IP)

Cells were transfected with appropriate plasmids using Lipofectamine 2000 reagent (Invitrogen) or by electroporation using a Microporator (Disital Bio). The total amounts of plasmid DNAs were standardized by addition of an empty vector. For immunoprecipitation (IP), cells were solubilized in 200 μl of IP lysis buffer (50 mM HEPES-KCl [pH 7.5], 150 mM NaCl, 0.5% TritonX100, 10% Glycerol, 2 mM EDTA and protease inhibitor mixture [Roche]). Cell extracts were then diluted with 800 μl of IP lysis buffer. Where indicated, 250U of Benzonase (Novagen) was added into the mixture and incubated 90 min at 25 °C. After cell lysates were precleared, supernatants were then mixed with either anti-FLAG M2 antibody or anti-HA antibody and then incubated at 4 °C for 3 h with rotation. Immunocomplexes were washed five times with IP lysis buffer. Samples were subjected to SDS-PAGE, followed by immunoblotting with the antibodies indicated in the figures and figure legends. We used TrueBlot anti-rabbit/mouse IgG HRP-conjugated antibodies (eBioscience) as secondary antibodies to eliminate the immunoglobulin heavy chain/light chain-specific band.

### Expression plasmids and mutagenesis

The expression vector for BALF5 was made by inserting the BALF5 open reading frame into pcDNA3.1(−) (Invitrogen). Mutant vectors were generated by a PCR-based method. Primers used in constructing mutant vectors are indicated in Supplemental Table 1. Plasmids for HA-tagged BSLF1 (HA-BSLF1), HA-BBLF2/3 and HA-BBLF4 were produced by inserting each ORF into EcoRI/HindIII site of pcDNA3.1(−), respectively. The primers used for these constructs were also shown in Supplemental Table 2.

### Cell-based polymerase complementation assays

*In vivo* polymerase complementation assays were carried out as described previously^8^. In brief, 50 ng of indicated expression plasmids were transfected into HEK293 EBV-BAC BALF5Δ cells. After 24 h, aliquots of cells were harvested and subjected to SDS-PAGE and immunoblotting to assess the protein expression levels. To measure viral genome amplification, quantitative real-time PCR assays (qPCR) were performed 48 h post-transfection, as documented previously^8^.

### *In vitro* transctiption/translation and DNA polymerase assays

EBV Pol proteins (BALF5) and/or polymerase processivity factor protein (BMRF1) were expressed using the Quick Coupled Transcription/Translation Systems (Promega) approach according to the manufacturer’s instructions with slight modifications^22^. In brief, 1 μg of plasmids were mixed with 20 μM of cold methionine, TNT quick master mix and nuclease-free water to a final volume of 50 μl. The mixture was incubated at 30 °C for 90 min. Protein expression levels were determined with SDS-PAGE and immunoblotting. Aliquots of mixture were subjected to DNA polymerase assays as described previously^10^,^12^,^13^. The mixtures were supplemented with a polymerase reaction buffer composed of 50 mM Tris-HCl (pH 7.5), 100 mM (NH_4_)_2_SO_4_, 50 μg/ml bovine serum albumin, 1 mM dithiothreitol (DTT), 3 mM MgCl_2_, 10 μg/ml activated calf thymus DNA, which was prepared by DNase I treatment^23^, 5 μM (each) dATP, dTTP, and dGTP, and 50 nM dCTP and [α-^32^P]dCTP. The reaction mixture was incubated at 37 °C and aliquots were removed at the indicated time points, mixed with EDTA and incubated on ice to stop further enzymatic activity. Samples were spotted on DE81 anion-exchange filters (Millipore) and washed twice with 5% (wt/vol) Na_2_HPO_4_ buffer for 5 min, rinsed once with deionized water for 5 min, and with 100% ethanol for 5 min. After the filter was air dried, aliquots of reaction mixture were dotted as a control. The incorporated [α-^32^P]dCMP was measured with the BAS-2500 system (Fuji Film).

### Genetic manipulation of EBV-BAC DNA

EBV-BAC DNA was provided by W. Hammerschmidt^24^. Homologous recombination was undertaken in *E. coli* as described previously^21^,^25^ with the oligonucleotide primers listed in Supplemental Figures. Electroporation was performed using a Gene Pulser III (Bio-Rad), and purification of EBV-BAC DNA was achieved with NucleoBond Bac100 (Macherey-Nagel, Germany).

### Immunofluorescence analysis

Immunofluorescence analysis was performed basically as described previously^17^. All staining procedures except for extraction and incubation with primary antibodies were carried out at room temperature. The primary antibodies were employed at a 1:200 (FLAG) and 1:300 (BALF2) dilution, respectively, and the secondary antibodies were employed at a 1:500 dilution. All washes after antibody incubation were performed with PBS containing 0.1% normal goat serum and 0.01% Tween 20. The slides were mounted in ProLong Gold antifade reagent with 4’,6’-diamidino-2-phenylindole (DAPI) (Molecular Probes) and analyzed by fluorescence confocal microscopy. Laser scanning confocal fluorescence microscopic images were captured and processed using an LSM510 Meta microscope (Carl Zeiss MicroImaging, Inc.) with a plan-Apochromat 100**×**/1.4-numericalaperture oil immersion objective lends.

## Additional Information

**How to cite this article**: Narita, Y. *et al.* A Herpesvirus Specific Motif of Epstein-Barr Virus DNA Polymerase Is Required for the Efficient Lytic Genome Synthesis. *Sci. Rep.* **5**, 11767; doi: 10.1038/srep11767 (2015).

## Supplementary Material

Supplementary Information
