# Supplementary material for: A Herpesvirus Specific Motif of Epstein-Barr Virus DNA Polymerase Is Required for the Efficient Lytic Genome Synthesis
Source: Sci Rep. 2015 Jun 30;5:11767. doi: 10.1038/srep11767 (PMC4485236; doi:10.1038/srep11767)
Supplement: Supplementary Information [file srep11767-s1.pdf]

Supplemental Information

A Herpesvirus Specific Motif of Epstein-Barr Virus DNA Polymerase Is Required for the Efficient Lytic Genome Synthesis

Yohei Narita[1, 2], Atsuko Sugimoto[1, 2], Daisuke Kawashima[1], Takahiro Watanabe[2], Teru Kanda[3], Hiroshi Kimura[2], Tatsuya Tsurumi[1] and Takayuki Murata[1, 2]*

[1]Division of Virology, Aichi Cancer Center Research Institute, 1-1 Kanokoden, Chikusa-ku, Nagoya 464-8681, Japan.

[2]Department of Virology, Nagoya University Graduate School of Medicine, 65 Tsurumai-cho, Showa-ku, Nagoya 466-8550, Japan.

[3]Division of Microbiology and Oncology, Aichi Cancer Center Research Institute, 1-1 Kanokoden, Chikusa-ku, Nagoya 464-8681, Japan.

Supplemental Figure 1

a

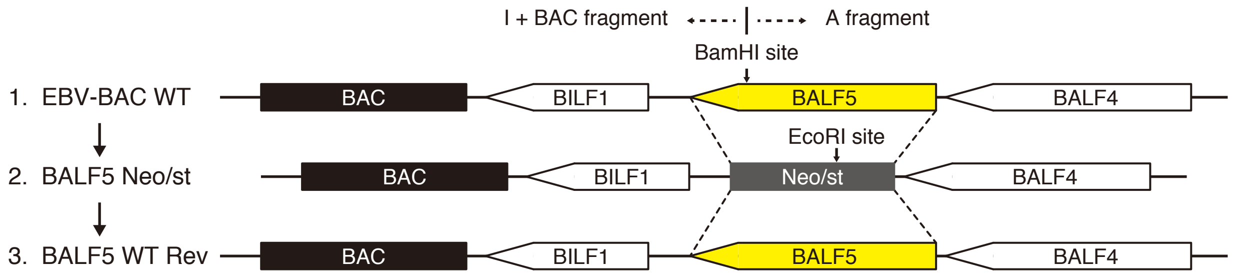

b

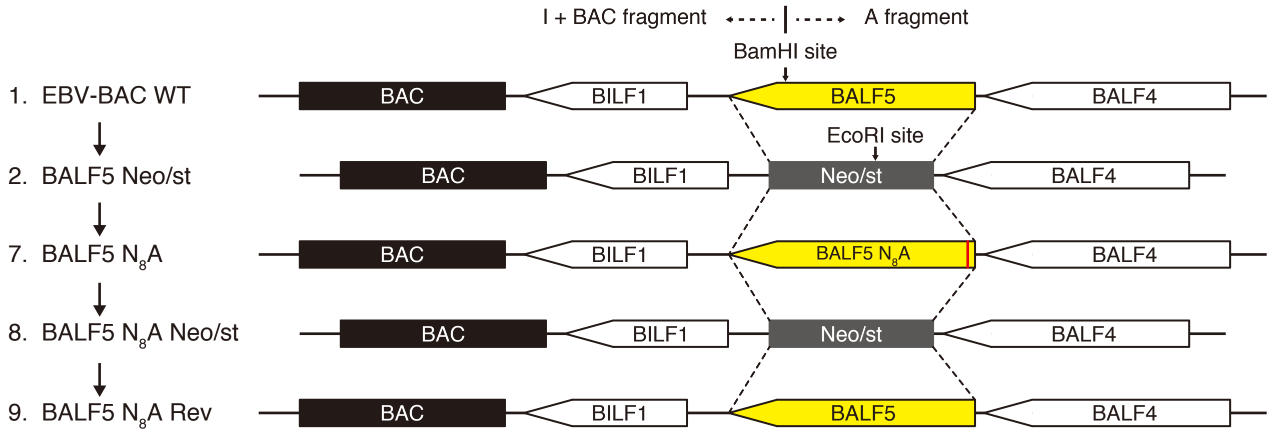

Supplemental Figure 2

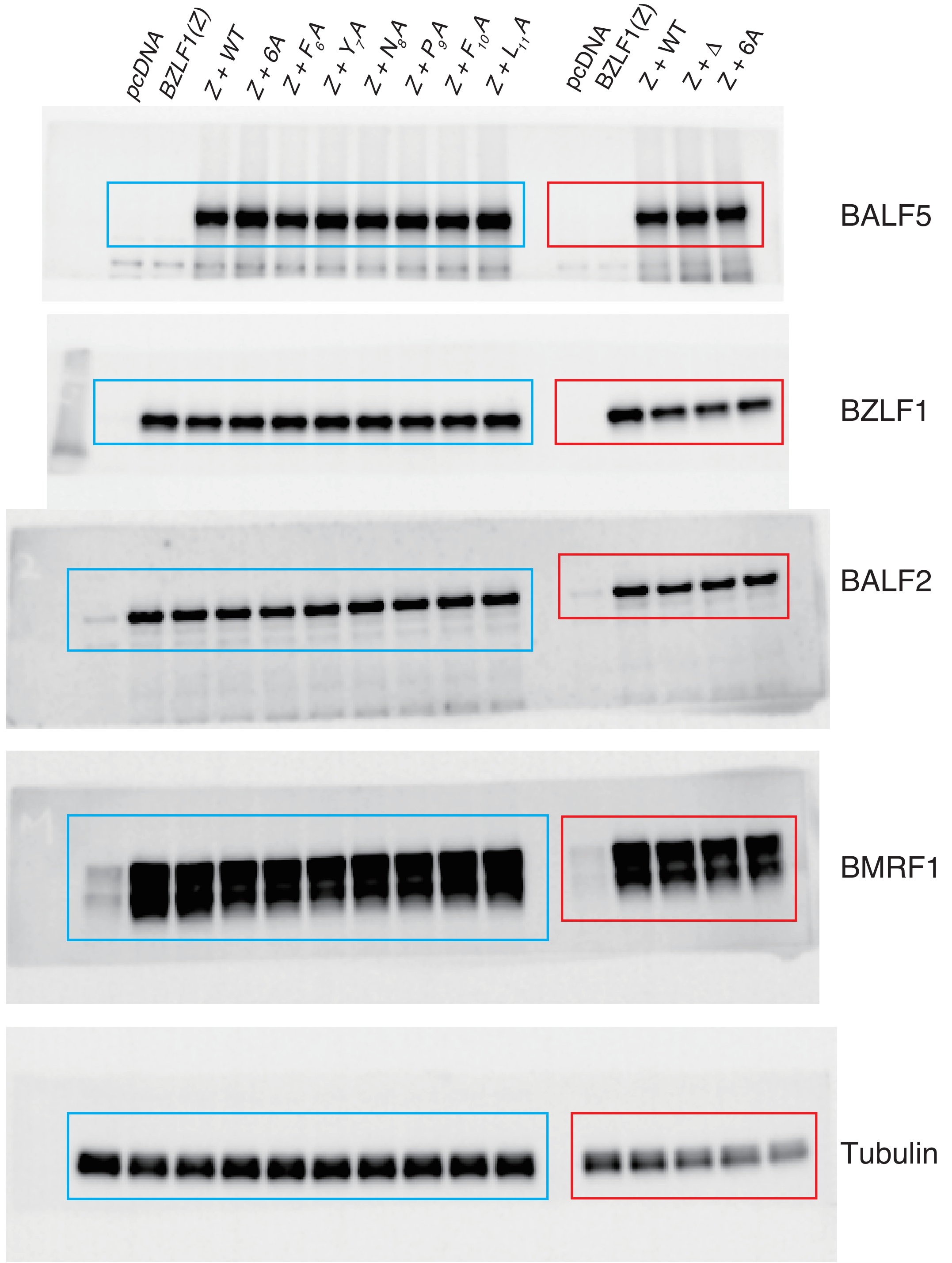

* Images surrounded by [red box] were used for Fig. 1c

* Images surrounded by [blue box] were used for Fig. 1f

Supplemental Figure 3

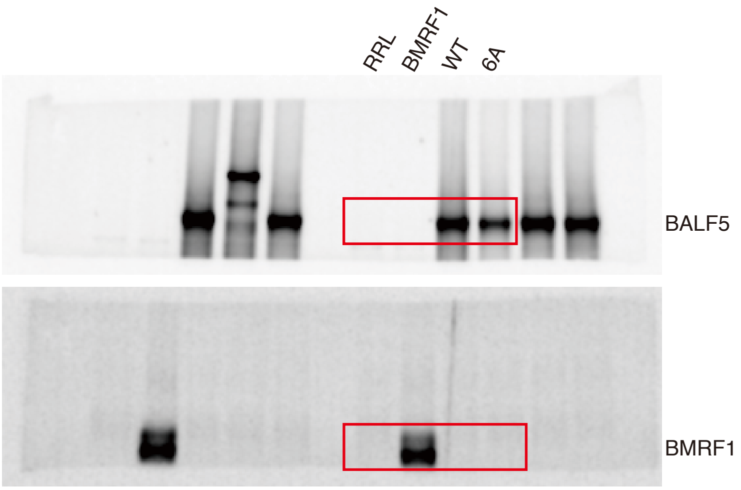

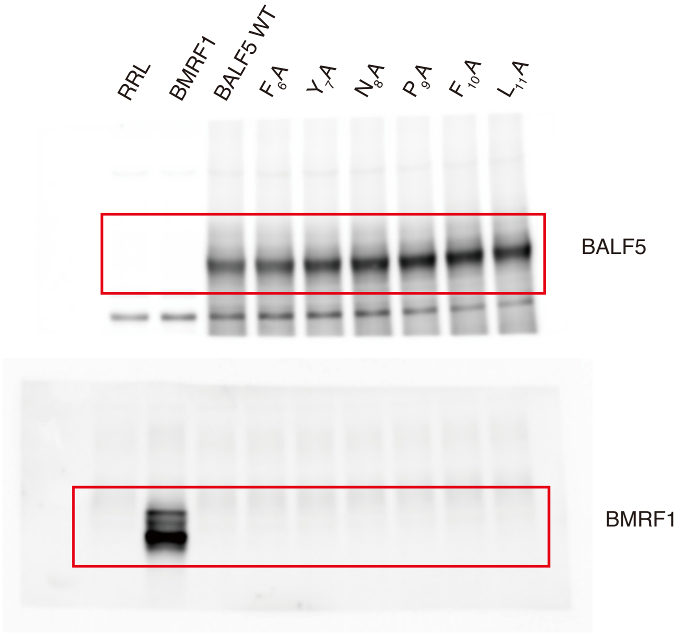

Supplemental Figure 4

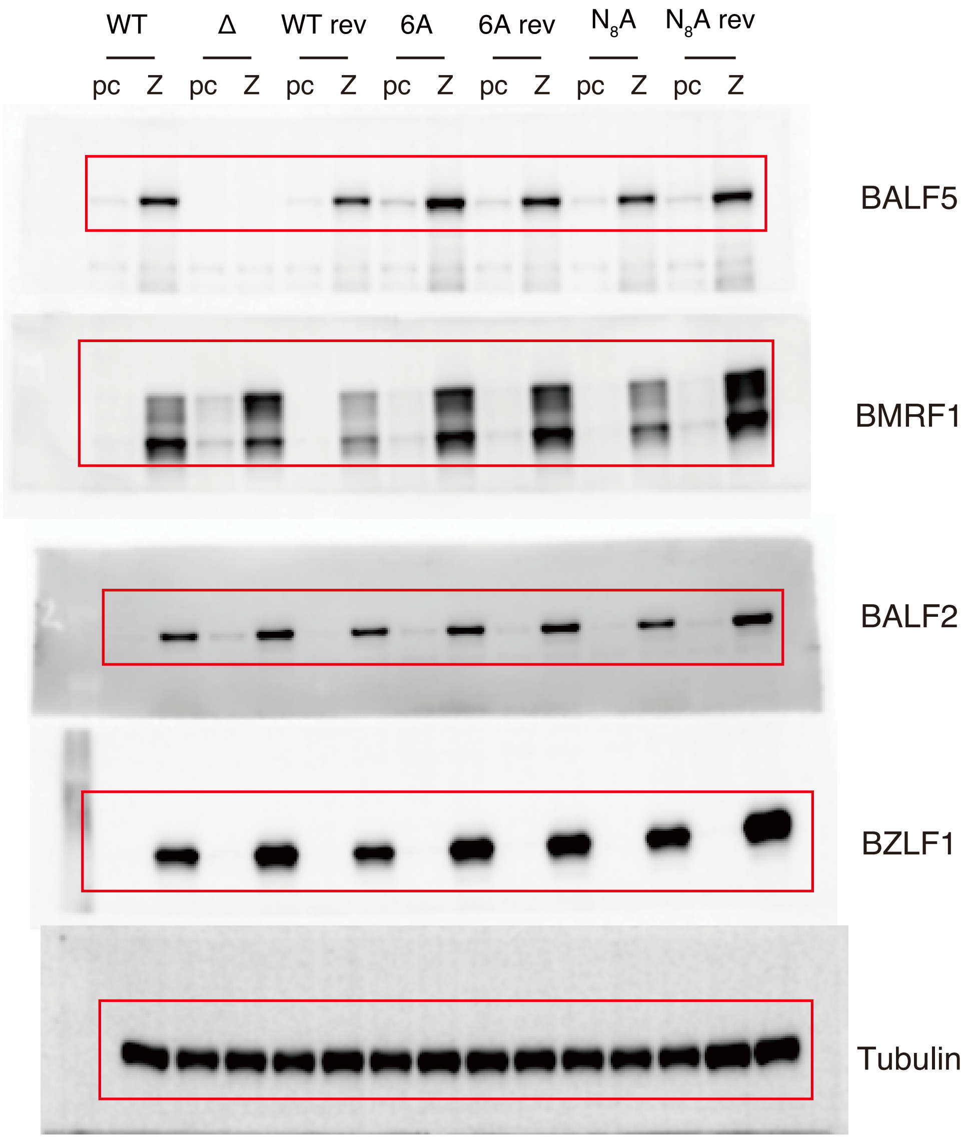

Supplemental Figure 5

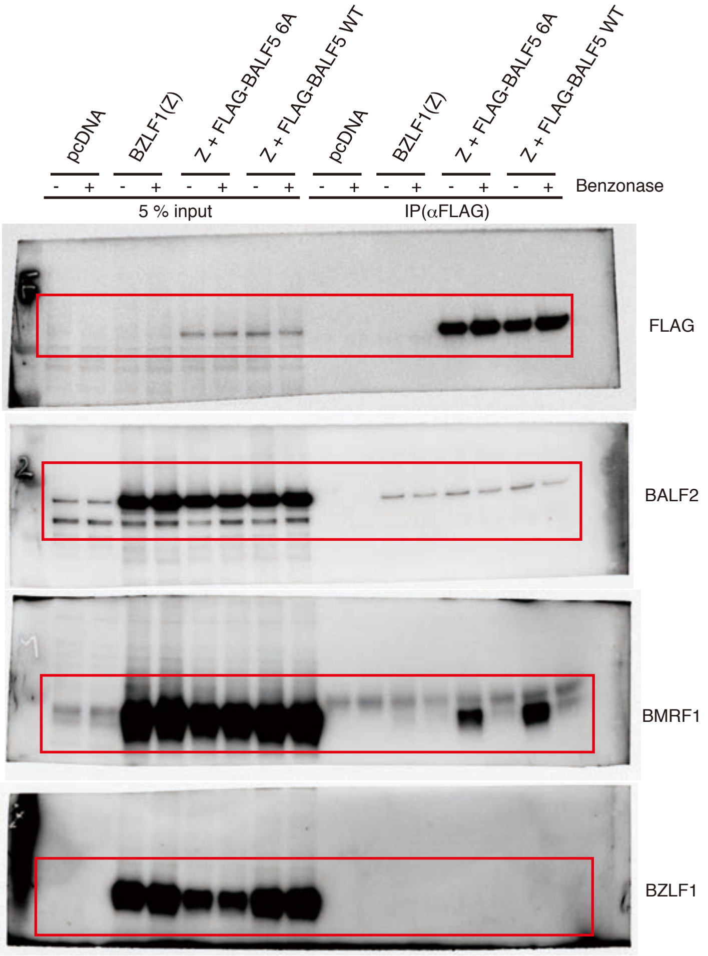

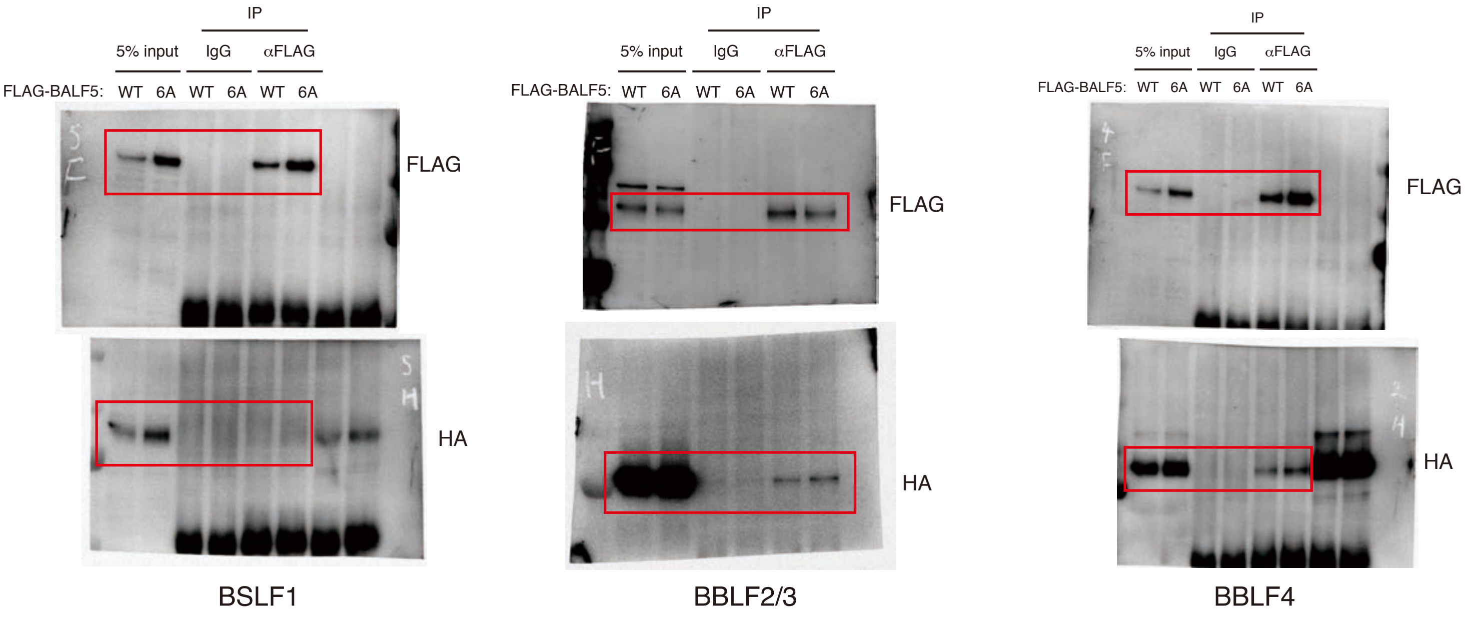

Supplemental Table 1

Oligonucleotide Primers used for constructing plasmids.

Primer name	Sequence (5' to 3')
BALF5 ΔFYNPFL Fw	AGACCTAATAAAGGCCTTC
BALF5 ΔFYNPFL/F_6A Rv	GAGTCCCCCAGACATGAATTC
BALF5 6A Fw	GCGGCAGCGAGACCTAATAAAGGCCTTC
BALF5 6A Rv	AGCGGCAGCGAGTCCCCCAGACATGAATTC
BALF5 F_6A Fw	GCCTATAACCCTTTCCTAAGAC
BALF5 Y_7A Fw	GCTAACCCTTTCCTAAGACCTA
BALF5 Y_7A Rv	GAAGAGTCCCCCAGACATG
BALF5 N_8A Fw	GCACCTTTCCTAAGACCTAATAAAG
BALF5 N_8A Rv	ATAGAAGAGTCCCCCAGAC
BALF5 P_9A Fw	GCTTTCCTAAGACCTAATAAAG
BALF5 P_9A Rv	GTTATAGAAGAGTCCCCCAG
BALF5 F_{10}A Fw	GCACTAAGACCTAATAAAGGCC
BALF5 F_{10}A Rv	AGGGTTATAGAAGAGTCCC
BALF5 L_{11}A Fw	GCAAGACCTAATAAAGGCCTTCTG
BALF5 L_{11}A Rv	GAAAGGGTTATAGAAGAGTC
HA-BSLF1 Fw	GCGCGGAATTCATGTATCCATATGACGTTCCAGATTACGCTTCC GCCCCCGTCGTCATCAAG
HA-BSLF1 Rv	GCGCGAAGCTTCTAGTTCGGGAGAGTCTCTGAG
HA-BBLF2/3 Fw	GCGCGGAATTCATGTATCCATATGACGTTCCAGATTACGCTATG GAAACACCCGCGGAGAGC
HA-BBLF2/3 Rv	GCGCGAAGCTTCTAGAATAAACTGAGAACAG
HA-BBLF2/3 Δintron Fw	GCTTTCCTGCAAGGCGTGAAAG
HA-BBLF2/3 Δintron Rv	CTGTGCTAGTGGCTCCTCTGAAG
HA-BBLF4 Fw	GCGCGGAATTCATGTATCCATATGACGTTCCAGATTACGCTGC CGAGGAGCCGAGGGCGCC
HA-BBLF4 Rv	GCGCGAAGCTTTCAGTAAACCAGTAGTGCGC
BSLF1 seq check	TCGTGCGCTACGTCTATTTG
BBLF2/3 seq check	TTCCTCCGCAGTATCTATGC
BBLF4 seq check	TTCGCGAGTGCGACAACATC

Supplemental Table 2

Oligonucleotide Primers used for generation of recombinant EBV.

**Primer name**	**Sequence (5' to 3')**
Neo/st Forward	CTATCACGATCCAGAGACCGCCGCCGCACTGCTTGGGGAGGC AGAGACTGAGTTTTAACCGGCCTGGTGATGATGGCGGGATC
Neo/st Reverse	TTCTCTCGTTTAAACGAGAGAATAGTAGTAGGGTCCAGTCTCA GGCCCCCTCACTTTGGGTCTCAGAAGAACTCGTCAAGAAGG
Forward transfer vector for 6A	CTATCACGATCCAGAGACCGCCGCCGCACTGCTTGGGGAGGC AGAGACTGAGTTTTAACCATGTCTGGGGGACTCGCTGCCGC
Forward transfer vector for N_8A	CTATCACGATCCAGAGACCGCCGCCGCACTGCTTGGGGAGGC AGAGACTGAGTTTTAACCATGTCTGGGGGACTCTTCTATGC
Forward transfer vector for wild type and revertant	CTATCACGATCCAGAGACCGCCGCCGCACTGCTTGGGGAGGC AGAGACTGAGTTTTAACCATGTCTGGGGGACTCTTCTATAAC
Reverse transfer vector for all	TTCTCTCGTTTAAACGAGAGAATAGTAGTAGGGTCCAGTCTCA GGCCCCCTCACTTTGGGTCTTAGAATGGTGGCCGGGCTG

Supplemental Table 3

Comparisons of DNA levels among EBV Pol mutants. (WT = 100%)

a

Figure 1d, 48h

pcDNA	0.980%
BZLF1 (Z)	1.39%
Z + WT	100%
Z + Δ	1.23%
Z + 6A	1.25%

b

Figure 1g, 48h

pcDNA	1.78%
BZLF1 (Z)	1.76%
Z + WT	100%
Z + 6A	1.42%
Z + F_6A	35.2%
Z + Y_7A	28.6%
Z + N_8A	2.05%
Z + P_9A	33.3%
Z + F_{10}A	30.8%
Z + L_{11}A	20.2%

c

Figure 3e

WT	100%
Δ	1.87%
WT rev	64.1%
6A	1.59%
6A rev	85.3%
N_8A	2.97%
N_8A rev	128%
